# The anti-inflammatory activities of ethanol extract from Dan-Lou prescription *in vivo* and *in vitro*

**DOI:** 10.1186/s12906-015-0848-4

**Published:** 2015-09-09

**Authors:** Li-Na Gao, Xin Zhou, Yi Zhang, Yuan-Lu Cui, Chun-Quan Yu, Shan Gao

**Affiliations:** Research Center of Traditional Chinese Medicine, Tianjin University of Traditional Chinese Medicine, YuQuan Road, Tianjin, P R China; Tianjin State Key Laboratory of Modern Chinese Medicine, Tianjin University of Traditional Chinese Medicine, Yu Quan Road, Tianjin, P R China; Tianjin University of Traditional Chinese Medicine, Tianjin, P R China

## Abstract

**Background:**

Although, Dan-Lou prescription (DLP) is used for antagonizing check discomfort and heartache, the pharmacological mechanism has not been clearly illustrated. Our present study aimed to design inflammatory models induced by LPS *in vivo* and *in vitro* to investigate the anti-inflammation of DLP ethanol extract (EEDL) and the potential mechanisms.

**Methods:**

EEDL was prepared and then analyzed by high performance liquid chromatography (HPLC). Further, the anti-inflammatory effects of EEDL *in vivo* was evaluated by measuring inflammation-associated factors includingcytokines, chemokines and acute phase proteins in lipopolysaccharide (LPS)-induced mice serum and liver. The anti-inflammatory mechanism exploration of EEDL was performed in LPS-stimulated RAW 264.7 cells. Different effects of EEDL on nitric oxide (NO) and prostaglandin (PG)E_2_ secretion were investigated by Griess reagent method and enzyme-linked immunosorbent assay (ELISA) respectively. Then the mRNA and protein expression of inducible NO synthase (iNOS) and cyclooxygenase (COX)-2 were measured by real-time reverse-transcription polymerase chain reaction (RT-PCR), ELISA and Western blot. Other chemokines and acute phase proteins were determined by proteome profile array. Finally, the ELISA based transcription factor assay was applied to measure the DNA-binding activity of nuclear transcription factor (NF)-κB p65.

**Results:**

Eight compounds from EEDL have been identified as gallic acid, salvianic acid, puerarin, daidzin, paeoniflorin, salvianolic acid B, cryptotanshinone, and tanshinone IIA, with amounts of 0.26, 9.84, 10.41, 2.55, 9.44, 3.82, 0.24 and 0.3 mg/kg, respectively. *In vivo*, EEDL administration antagonized the up-regulation of more than 17 kinds of cytokines, chemokines and acute phase proteins in LPS-treated mice serum, and inhibited LPS-induced IL-6 mRNA and protein expression in mice liver tissue. *In vitro*, LPS-induced NO and PGE_2_ over-productions were decreased by EEDL treatment. The mRNA and protein expression of iNOS, COX-2 and IL-6 were similarly inhibited by EEDL treatment, which might be attributed to decrease the DNA-binding activity of NF-κB p65.

**Conclusion:**

EEDL was valid for anti-inflammation and the potential molecular mechanisms might be due to the inhibition of of LPS-induced iNOS/NO, COX-2/PGE_2_ and cytokines expression by antagonizing the activation of NF-κB p65.

**Electronic supplementary material:**

The online version of this article (doi:10.1186/s12906-015-0848-4) contains supplementary material, which is available to authorized users.

## Background

Coronary heart disease (CHD) is a common and frequently-occurring disease [1]. There are more than 3.8 million men and 3.4 million women die of the CHD each year and have a tendency to increase in recent years globally [2]. In clinical, Dan-Lou prescription (DLP) is given to patients with angina pectoris, and has advantages in the improvement of sputum, the lip empurples, etc. [3–5]. Researchers indicate that the pharmacological effects are attributed to increasing coronary flow [6], antagonizing platelet aggregation [7], thrombosis formation [8], hypertention [9], hyperglycemia and hyperlipidemia [10].

DLP is derived from the traditional Chinese medicine formula Gualou Xiebai Banxia Tang which is recorded in the Synopsis of Prescriptions of the Golden Chamber (Jin-Gui-Yao-Lue) and has been used for about 2000 years in China. DLP is consisted of 10 herbs, including *Trichosanthes kirilowii* Maxim. (Cucurbitaceae), *Allium macrostemon* Bunge. (Liliaceae), *Pueraria lobata* (Willd.) Ohwi (Leguminosae), *Ligusticum chuanxiong* Hort. (Umbelliferae), *Salvia miltiorrhiza* Bunge. (Labiatae), *Paeonia lactiflora* Pall. (Ranunculaceae), *Alisma plantago-aquatica* Linn. (Alismataceae), *Astragalus membranaceus* (Fisch.) Bunge. (Leguminosae), *Davallia mariesii* Moore ex Bak. (Davalliaceae), and *Curcuma aromatica* Salisb. (Zingiberaceae). To further investigate the chemical composition and pharmacological mechanism, numerous detection methods have been established such as liquid chromatography-mass spectrometry (LC-MS), gas chromatography–mass spectrometry (GC-MS), high performance liquid chromatography-chemiluminescence detection (HPLC-CL), high performance liquid chromatography-diode-array detector (HPLC-DAD) and ultra high performance liquid chromatography/diode-array detector/quadrupole time-of flight tandem mass spectrometry (UPLC-DAD-QTOF) [7, 11–14]. In this study, eight highest level ingredients of DLP ethanol extract (EEDL), gallic acid, salvianic acid, puerarin, daidzin, paeoniflorin, salvianolic acid B, cryptotanshinone and tanshinone IIA, were chosen as marker substances to investigate pharmacological mechanism.

More and more studies show that inflammation plays a vital role in CHD [15]. Atherosclerosis acts as the main cause of CHD and is formerly considered as a bland lipid storage disease. However, atherosclerosis is actually a process of inflammation invasion. In the pathological processes of atherosclerosis, all stages such as initiation, growth and complication of atherosclerotic plaque involve an inflammatory response [16]. The circulating inflammatory markers such as C-reactive protein (CRP), serum amyloid A (SAA), interleukin (IL)-6 and IL-1 receptor commonly accompany with acute coronary syndromes [17]. Pharmacological studies demonstrate that many active components of DLP such as gallic acid [18], puerarin [19], paeoniflorin [20], cryptotanshinone [21] and tanshinone IIA [22] have anti-inflammatory effects by antagonizing over-expression cyclooxygenase (COX)-2, inducible nitric oxide synthase (iNOS), IL-6, etc. In detail, gallic acid inhibits lipopolysaccharide (LPS)-induced inflammation in nuclear transcription factor (NF)-κB transgenic mice [18], cryptotanshinone suppresses LPS-induced mice cytokines secretion via inhibition of NF-κB [21], tanshinone IIA reduces LPS-induced mice acute lung injury via NF-κB inhibition [22]. For these facts, propose new strategies from anti-inflammation may be a new viewpoint for the prediction, prevention, and treatment of DLP on CHD.

The exploration of EEDL on inflammatory response is the most fundamental research to illustrate the pharmacological mechanisms. LPS-induced inflammatory models in mice and macrophages are widely used to preliminarily screen anti-inflammatory drugs [23]. In the present study, we designed inflammatory models induced by LPS *in vivo* and *in vitro* to investigate the anti-inflammation of EEDL and the potential mechanisms.

## Methods

### Reagents

The drug powder of DLP was provided by Jilin Connell Medicine Co., Ltd (Jilin, China). Dulbecco’s modified Eagle’s medium-high glucose (DMEM), 3-(4, 5-dimethylthiazol-2-yl)-2, 5-diphenyltetrazolium bromide (MTT), aspirin and LPS from *Escherichia coli* 0111:B4 were obtained from Sigma-Aldrich Co. (St. Louis, USA). IL-6 Mouse ELISA Kit was obtained from eBioscience (San Diedo, USA). Mammalian Cell Lysis Kit and UNIQ-10 column Trizol total RNA extraction kit were obtained from Sangon Biological Engineering Technology & Services Co., Ltd (Shanghai, China). Improm-II Reverse Transcription System was purchased from Promega Corporation (Madison, USA). FastStart Universal SYBR Green Master (ROX) kit was purchased from Roche (Mannheim, Germany). BCA Protein Assay Kit was purchased from Thermo Fisher (Rochford, USA). Dexamethasone injection was obtained from Shanghai General Pharmaceutical Co., Ltd. (Shanghai, China). 4-amino-5-methylamino-2′, 7′-difluorofluorescein diacetate (DAF-FM-DA) was obtained from Invitrogen (Carlsbad, USA). Prostaglandin (PG)E_2_ EIA monoclonal kit and Mouse Cytokine Array Panel A Array kit were obtained from R&D Systems, Inc. (Minneapoils, USA). Mouse anti-cyclooxygenase (COX)-2 monoclonal antibody was from BD PharMingen (San Diego, USA) and goat anti-Mouse IgG peroxidase conjugate was from Merck (Darmstadt, Germany). BAY 11–7082 and Nuclear and Cytoplasmic Protein Extraction Kit were purchased from Beyotime Institute of Biotechnology (Haimen, China). Antibody for iNOS was purchased from Cell Signaling Technology (Beverly, USA). Goat anti-rabbit IgG peroxidase conjugate was bought from Merck (Darmstadt, Germany). Transcription Factor Assay Kit for nuclear transcription factor (NF)-κB p65 was purchased from Thermo Fisher (Rochford, USA).

### Extract preparation

The DLP drug powder (50 g) was extracted with 75 % ethanol (500 mL) in an ultrasonic bath for 30 min at room temperature and then filtrated. The extraction process above mentioned was repeated for three times, mixed the filtrates and concentrated to 100 mL under reduced pressure at 60 °C. Finally, the concentrate was lyophilized, sealed and stored lucifugally in 50 mL centrifuge tube at 4 °C for use. The EEDL was dissolved in DMEM to obtain the desired concentrations in experiments.

### Quantitative chromatographic analysis

To ensure the consistent results, the constituents of EEDL were analyzed by HPLC using a Waters 2695 system (Waters, New York, USA), which is equipped with a quaternary pump, an autosampler, an online vacuum degasser, and an ultraviolet detector. The chromatographic analyses were performed on a Kromasil C18 Column (250 mm × 4.6 mm, 5 μm, Sweden) at 25 °C. For HPLC analysis, a 5 μL sample was injected into the column with a constant flow rate of 1.0 mL/min. The mobile phase consisted of acetonitrile (A) and water (containing 0.1 % formic acid, v/v, B). The elution was carried out as follows: 10–11 % A at 0–6 min, 11 % A at 6–8 min, 11–18 % A at 8–10 min, 18–33 % A at 10–17 min, 33 % A at 17–20 min, 33–52 % A at 20–21 min, 52–55 % A at 21–25 min, 55–75 % A at 25–26 min, 75–90 % A at 26–35 min, 90 % A at 35–40 min, 90–100 % A at 40–40.1 min. Chromatograms were monitored at a wavelength of 280 nm [7].

### *In vivo* anti-inflammatory analysis

#### Animals and acute inflammatory injury

Male ICR mice (18–22 g) were obtained from Beijing HuaFuKang Bio-technology Co., Ltd. They were group-housed in cages at constant humidity (55 ± 5 %), temperature (24 ± 1 °C) and light–dark cycle (on at 7:00 a.m. and off at 7:00 p.m.). All animals were allowed *ad libitum* water according to the experimental protocols. Studies were performed in accordance with NIH Guide for the Care and Use of Laboratory Animals and protocol was approved by the Ethics Committee of Tianjin University of Traditional Chinese Medicine (TCM-2009-037-E12).

Forty-eight mice were randomly divided into 6 groups (8 mice per group): blank control, LPS (dissolved in sterile pyrogen-free saline solution, 1 mg/kg body weight), LPS + EEDL (0.25, 1 and 4 g/kg); positive control (LPS + dexamethasone injection, 10 mg/kg). Mice were firstly pretreated intraperitoneally (i.p.) with 0.9 % saline, different amount of EEDL and dexamethasone injection, which were dissolved in 0.9 % saline to make a total volume of 200 μL. After drug administration for 30 min, LPS were then i.p. given to mice for 90 min, while blank control mice received only 0.9 % saline. Subsequently, mice were anesthetized with ether and blood samples were collected. Serum was obtained by incubating for 1 h at 37 °C (water bath), and then centrifuged at 3000 rpm for 10 min at 4 °C and stored at −20 °C until use. Simultaneously, the liver tissues were quickly removed, frozen in liquid nitrogen immediately and stored at −80 °C until analysis.

#### Analysis of IL-6 protein and mRNA expression in liver

The IL-6 level in mice liver was detected according to the manufacturer’s instructions of commercially available ELISA kits. The protein was quantified by BCA method. For total RNA extraction, the mice tissues of liver samples (about 30 mg) were homogenized in 500 μL of Trizol reagent. Real-time RT-PCR was performed according to a previously described method [23]. The real-time RT-PCR oligonucleotide primers used for mouse IL-6 and β-actin (internal control) were shown in Table 1.

**Table 1:** Real-time RT-PCR oligonucleotide primers

Gene	Primer	Sequence (5′–3′)	PCR product (bp)
β-actin	Forward	TGTTACCAACTGGGACGACA	165
(NM_007393.3)	Reverse	GGGGTGTTGAAGGTCTCAAA	
iNOS	Forward	CACCTTGGAGTTCACCCAGT	170
(NM_010927.3)	Reverse	ACCACTCGTACTTGGGATGC	
COX-2	Forward	TGAGTACCGCAAACGCTTCTC	151
(NM_011198.3)	Reverse	TGGACGAGGTTTTTCCACCAG	
IL-6	Forward	TCCAGTTGCCTTCTTGGGAC	140
(NM_031168.1)	Reverse	GTGTAATTAAGCCTCCGACTTG	

#### Cytokine protein array analysis

To screen for different acute phase proteins, cytokines, and chemokines in mice serum, a protein profile array kit (Mouse Cytokine Array Panel A) from R&D Systems (cat.no.ARY006) was performed according to the manufacturer’s instructions. Samples of mice serum (150 μL) were separately run on the array [24–26]. The proteins were detected including: BLC, C5a, G-CSF, GM-CSF, I-309, Etoaxin, sICAM-1, IFN-γ, IL-1α, IL-1β, IL-1ra, IL-2, IL-3, IL-4, IL-5, IL-6, IL-7, IL-10, IL-13, IL-12p70, IL-16, IL-17, IL-23, IL-27, IP-10, I-TAC, KC, M-CSF, JE, MCP-5, MIG, MIP-1α, MIP-1β, MIP-2, RANTES, SDF-1, TARC, TIMP-1, TNF-α and TREM-1. Horseradish peroxidase chemiluminescent substrate (Millipore Corporation, USA) was used to detect protein blotting by exposure to Kodak BioMax Light films. Films were scanned into a computer and densitometry of the image was performed using an Image-Pro Plus Software version 6.0 (Media Cybernetics, Sliver Spring, MD, USA).

### *In vitro* anti-inflammatory activities

#### Cells and cell culture

The murine macrophage cell line RAW 264.7 was obtained from Cell Culture Center of Chinese Academy of Medical Sciences (Beijing, China). Cells were cultured in DMEM supplemented with 10 % HI-FBS, 100 U/mL penicillin and 100 μg/mL streptomycin at 37 °C in a fully humidified incubator containing 5 % CO_2_. For all experiments, cells were grown to a confluence of 80–90 %, and were subjected to no more than seven cell passages.

#### Drug dilution

EEDL was prepared as above mentioned. Then a series of concentrations (200, 100, 50 and 25 μg/mL) were obtained by diluting with DMEM (without phenol red) which was supplemented with 5 % heat inactivated fetal bovine serum (HI-FBS) and 0.2 μg/mL LPS.

### Analysis of cell viability

RAW 264.7 cells were distributed into 96-well plates (1.0 × 10^4^ cells per well) for 24 h, and treated with various concentrations of EEDL (200, 100, 50, and 25 μg/mL), L-NAME (100 μM), and aspirin (100 μM). In this assay, both L-NAME and aspirin work as positive control drugs. L-NAME is an inhibitor of NOS, and aspirin is a commonly used anti-inflammatory drug which prevent the transformation of arachidonic acid to both PGE and thromboxanes by inhibiting COX activity. After incubated for 20 h, MTT reagent was added to a final concentration of 0.5 mg/mL and incubated for an additional 2 h at 37 °C and 5 % CO_2_. After discarding the medium, the formazan precipitate was solubilized in 100 μL DMSO, and the absorbance was recorded at 570 nm using a multifunctional microplate reader (FlexStation 3, Molecular Devices, USA).

### Determination of nitrite secretion, intracellular NO production, iNOS mRNA and protein expression

Nitrite is the stable product from NO and is used as an indicator of NO secreted into the culture medium. RAW 264.7 cells, cultured in 96-well plates (5.0 × 10^4^ cells per well) overnight were pretreated with various concentrations of EEDL (200, 100, 50 and 25 μg/mL), L-NAME (100 μM) or aspirin (100 μM) containing 0.2 μg/mL LPS. The LPS-treated group and blank control group was given equal volume of medium with or without LPS (0.2 μg/mL), respectively. After 24 h, the nitrite concentration accumulated in the culture supernatants was monitored by Griess reagent system (Promega, USA). Intracellular NO was detected by using the NO-sensitive fluorescent dye DAF-FM-DA. In detail, the RAW 264.7 cells were seeded in 35-mm dishes for 24 h at 37 °C and 5 % CO_2_, and stimulated with EEDL (200 μg/mL) and L-NAME (100 μM) in the present of LPS (0.2 μg/mL) for an additional 24 h. Similarly, LPS-treated group and blank control group was given equal volume of medium with or without LPS (0.2 μg/mL), respectively. Subsequently, the culture medium was discarded and cells were fixed with 4 % paraformaldehyde. Finally, 10 μM DAF-FM-DA was incubated for 1 h and washed with PBS (pH 7.4) for three times. Digitized images were picked with a Zeiss LSM 710 confocal laser scanning microscope with an excitation wavelength of 495 nm and an emission wavelength of 515 nm. For the detection of iNOS mRNA expression, cells were collected from 6-well plates with or without the stimulation of LPS, and performed as earlier described [23].

### Assay for PGE_2_ production, COX-2 mRNA and protein expression

RAW 264.7 cells were treated with the indicated concentrations of EEDL (200, 100 and 50 μg/mL) containing 0.2 μg/mL LPS for 24 h, in addition to the LPS-treated group and blank control group which was given equal volume of medium with or without LPS (0.2 μg/mL), respectively. The cell supernatants were drawn off for the detection of PGE_2_ using the PGE_2_ EIA monoclonal kit (Minneapoils, USA), and cells were used to measure the expression of COX-2 protein with cell-based ELISA as previously reported [23, 27]. The COX-2 mRNA expression was conducted similarly as earlier described.

### Determination of IL-6 mRNA expression and protein secretion

To determine the level of IL-6 production in cell supernatants, cells were plated at 1 × 10^5^ cells per well in 48-well plate and stimulated with various concentrations of EEDL (200, 100 and 50 μg/mL) in the presence of 0.2 μg/mL LPS for an additional 24 h. Similarly, the LPS-treated group and blank control group was given equal volume of medium with or without LPS (0.2 μg/mL), respectively. For the detection of IL-6 mRNA, cells were cultured in 6-well plate with the density of 1 × 10^6^ cells per well. After stimulation with LPS, cells were harvested for real-time RT-PCR as earlier described [23].

### Cytokine protein array analysis and western blot

To screen for different acute phase proteins, cytokines, and chemokines in RAW 264.7 cell lysate, a total protein content of 20 μg was run on the array and performed as earlier described. Cell lysate was prepared with a Nuclear and Cytoplasmic Protein Extraction Kit (Beyotime Institute of Biotechnology, China) according to the manufacturer’s protocol. For the protein analysis of iNOS, cells were seeded in the 6-well plates at a density of 1 × 10^6^ cells per well. After stimulation with LPS for 24 h, cell lysate was prepared as above mentioned. Subsequently, BCA method was employed to determine the protein concentration, and equal amounts (40 μg) of protein were subjected to SDS-PAGE and boiled for 5 min. following transferring samples onto polyvinylidene fluoride (PVDF) membrane, TTBS (0.5 % Tween 20, 10 mM Tris–HCl, pH 7.5, 150 mM NaCl) containing 5 % nonfat milk was used for blocking at room temperature for 1 h. One hour later, blots were incubated with antibodies against iNOS (1:500), or β-actin (1:5000) overnight at 4 °C. After washing, appropriate secondary antibodies were selected for incubation at room temperature for another 1 h. Finally, the redundant secondary antibodies were washed and the protein bands were detected by a chemiluminescence detection kit (Millipore, Billerica, MA, USA) and exposure to Kodak BioMax Light films. After the films were scanned and saved as tagged image file format (TIFF) image files, images were quantified using Image-Pro Plus version 6.0 (Media Cybernetics, Sliver Spring, MD, USA).

### DNA-binding activity of NF-κB p65

RAW 264.7 cells, cultured in 60-mm dishes with a density of 1.5 × 10^6^ cells per well, were pretreated with EEDL (200, 100 and 50 μg/mL) or BAY 11–7082 for 2 h. BAY 11–7082 is a commonly used inhibitor of NF-κB*.* Then LPS (with a final concentration of 0.2 μg/mL) was added for an additional 30 min. The LPS-treated group and blank control group was given equal volume of medium with or without LPS (0.2 μg/mL), respectively. Then the cell lysate was prepared with a Nuclear and Cytoplasmic Protein Extraction Kit (Beyotime Institute of Biotechnology, China) according to the manufacturer’s protocol. For the DNA-binding activity of NF-κB p65, BCA method was applied for the quantification of protein concentration. Finally, 3 μg protein was added as the manufacture’s instruction of Transcription Factor Assay Kit (Thermo Fisher Scientific Inc., USA).

### Statistical analysis

The Origin 7.5 software (MicroCal, USA) was applied to perform statistical analysis. N indicated the number of animals in each group or the number of wells studied in each category. *In vivo* experiments, data were expressed as means ± SE, and *in vitro*, means ± SD were calculated. For statistical comparisons, the results were analyzed using one-way analysis of variance (ANOVA) and a *P* < 0.05 was considered statistically significant.

## Results

### Primary chemical ingredients analysis of EEDL

**Fig. 1:**
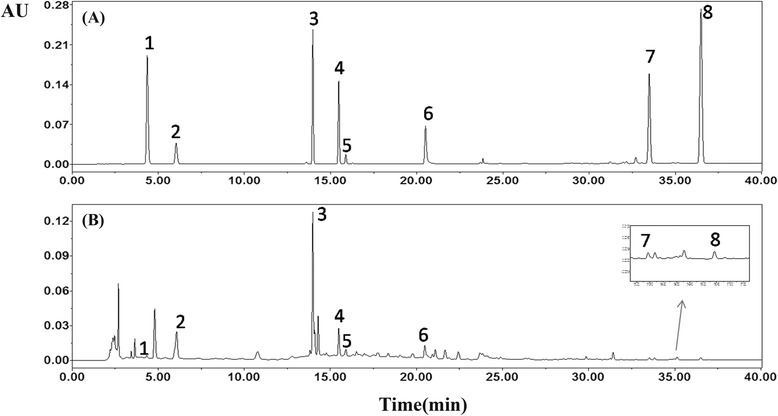
HPLC analysis of (**a**) standard solutions and (**b**) sample of EEDL. “1” gallic acid; “2” salvianic acid; “3” puerarin; “4” daidzin; “5” paeoniflorin; “6” salvianolic acid B; “7” cryptotanshinone; and “8” tanshinone IIA

Analysis of various chemical compounds in EEDL was performed by HPLC. The typical chromatogram of EEDL is shown in Fig. 1 and eight compounds from EEDL have been identified as gallic acid (1), salvianic acid (2), puerarin (3), daidzin (4), paeoniflorin (5), salvianolic acid B (6), cryptotanshinone (7), and tanshinone IIA (8). According to the calibration curves, the contents of the eight compounds above mentioned in EEDL were quantified. The amounts of gallic acid, salvianic acid, puerarin, daidzin, paeoniflorin, salvianolic acid B, cryptotanshinone, and tanshinone IIA were 0.26, 9.84, 10.41, 2.55, 9.44, 3.82, 0.24 and 0.3 mg/kg, respectively.

### Anti-inflammation of EEDL *in vivo*

#### EEDL attenuates the IL-6 mRNA and protein expression in liver

**Fig. 2:**
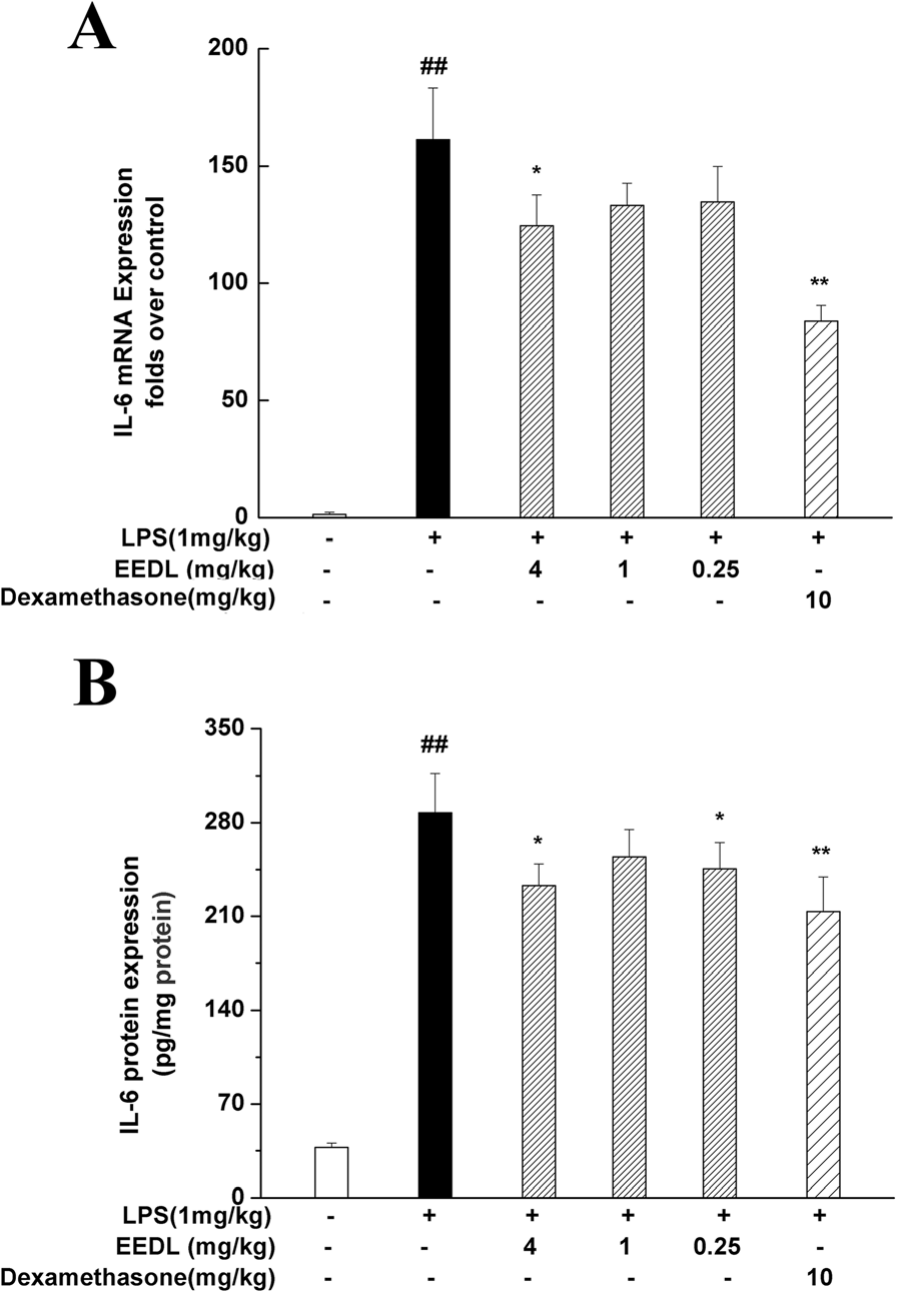
Effects of EEDL on LPS-induced IL-6 mRNA and protein expressions in mice liver. After mice were i.p. administrated with LPS (1 mg/kg) for 90 min, mice livers were selected for this assay. The mRNA and protein expressions of IL-6 (**a** and **b**) were measured with real-time RT-PCR and ELISA, respectively. Values are means ± SE (*n* = 3 for real-time RT-PCR, *n* = 6 for ELISA) and significant difference compared with blank control or LPS treated alone, ^##^ *P* < 0.01 *vs*. blank control group, *P < 0.05, **P < 0.01 *vs*. LPS-treated group

As a pro-inflammatory cytokine, IL-6 plays a vital role in the process of inflammation. When mice were i.p. injected with LPS (1 mg/kg), the IL-6 mRNA (Fig. 2a) and protein (Fig. 2b) level in mice liver were significantly increased up to hundreds folds comparing with the blank control group (*P <* 0.01). However, dexamethasone injection or EEDL (0.25, 1 and 4 mg/kg) treatment down-regulated the aberrant increase of IL-6 mRNA and protein expression with varying degrees (*P <* 0.05 or *P <* 0.01).

#### Effects of EEDL on multiple cytokines in serum

**Fig. 3:**
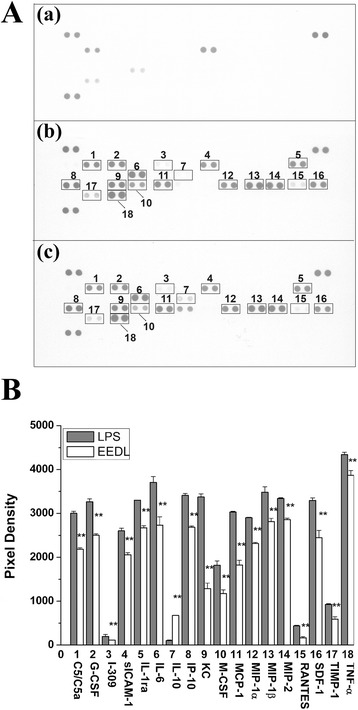
Effects of EEDL on LPS-induced multiple cytokines production in mice serum. After mice were pre-treated with EEDL (4 mg/kg) or 0.9 % saline for 30 min, LPS (1 mg/kg) or 0.9 % saline were injected, respectively, for an additional 90 min. **a** Expression of multiple proteins in the acute inflammation was measured by proteome profile array. (*a*) Blank control; (*b*) mice were administrated (i.p.) with LPS alone; (*c*) mice were administrated (i.p.) with LPS + EEDL. Numbers marked on the membranes are presented as: “1” C5/C5a; “2” G-CSF; “3” I-309; “4” sICAM-1; “5” IL-1a; “6” IL-6; “7” IL-10; “8” IP-10; “9” KC; “10” M-CSF; “11” MCP-1; “12” MIP-1α; “13” MIP-1β; “14” MIP-2; “15” RANTES; “16” SDF-1; “17” TIMP-1; “18” TNF-α. **b** Analysis of optical density was captured by the Image-Pro Plus version 6.0. Values are means ± S.D. and significant difference compared with LPS treated alone, **P < 0.01

Previously, we showed that the inflammation induced by LPS (1 mg/kg) in mice was an inflammatory network composed of cytokines, chemokines and so on [23]. To further investigate the anti-inflammation of EEDL, the proteome profiler array was employed. It allows a simultaneous analysis of 40 proteins in a single experiment. As shown in Fig. 3, LPS resulted in a burst of 17 inflammation-related proteins, including C5/C5a, G-CSF, I-309, sICAM-1, IL-1ra, IL-6, IP-10, KC, M-CSF, MCP-1, MIP-1α, MIP-1β, MIP-2, RANTES, SDF-1, TIMP-1 and TNF-α. Also, LPS decreased the expression of IL-10, an anti-inflammatory cytokine. However, EEDL administration (4 mg/kg) not only notably suppressed the over-expressions of C5/C5a, G-CSF, I-309, sICAM-1, IL-1ra, IL-6, IP-10, KC, M-CSF, MCP-1, MIP-1α, MIP-1β, MIP-2, RANTES, SDF-1, TIMP-1, and TNF-α (*P <* 0.01), but increased the IL-10 protein levels (*P <* 0.01).

### Anti-inflammation of EEDL *in vitro*

#### Cytotoxicity of EEDL on RAW 264.7 cells

The cytotoxicity of EEDL on RAW 264.7 cells was detected with MTT assay. Comparing with the blank control group, no significant variation of optical density was observed among each group (Additional file 1). It suggested that all concentrations of EEDL, L-NAME and aspirin used were not toxic to RAW 264.7 cells.

#### Effect of EEDL on NO production, iNOS mRNA and protein expression

**Fig. 4:**
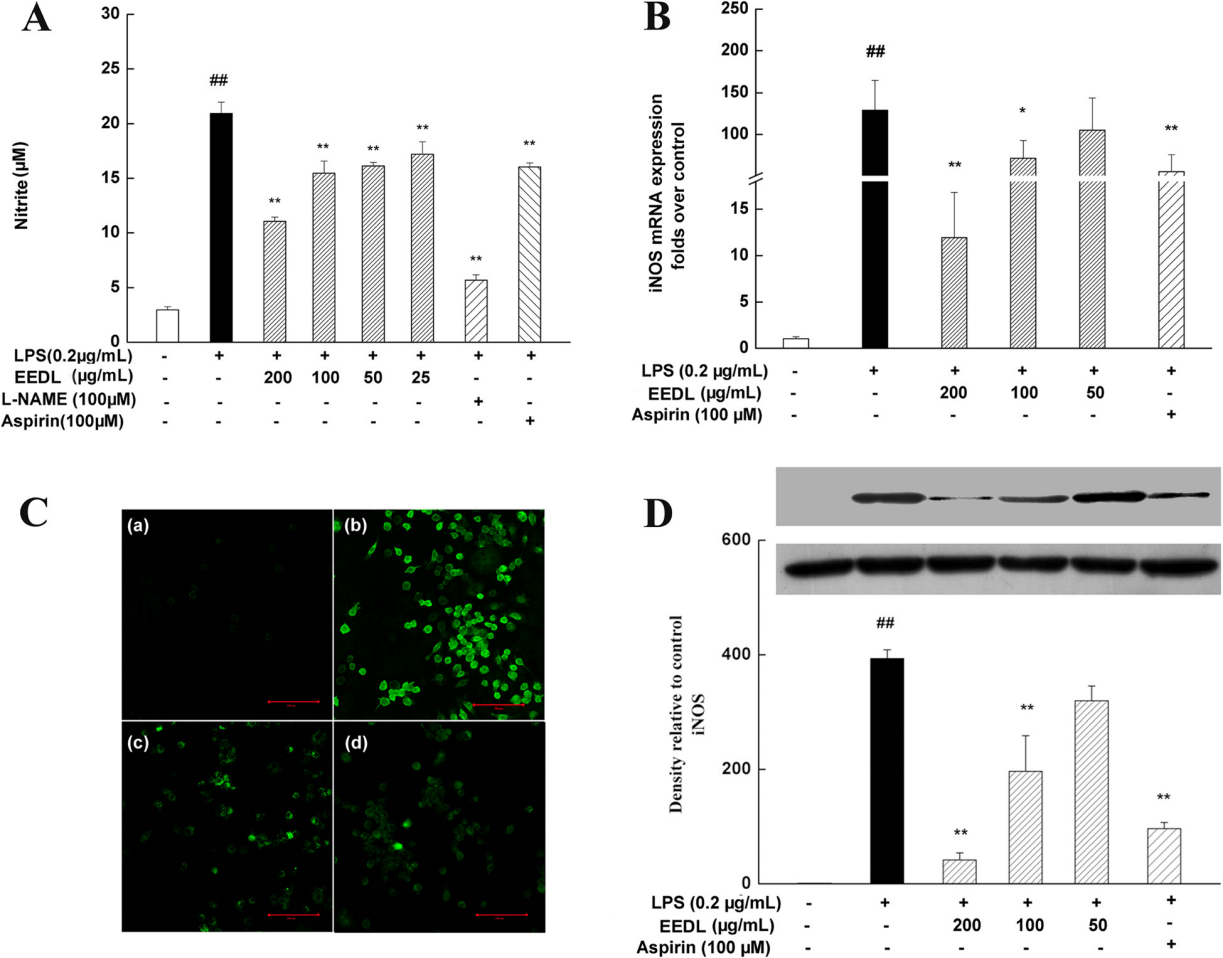
Effects of EEDL on NO and iNOS levels in LPS-stimulated RAW 264.7 cells. **a** Nitrite production secreted in the culture medium was monitored by the Griess reagent system. Values are means ± SD (*n* = 6) from three independent experiments and significant difference compared with blank control or LPS treated alone, ^##^ *P* < 0.01 *vs*. blank control group, **P < 0.01 *vs*. LPS-treated group. **b** RAW 264.7 cells were pretreated with EEDL (200 μg/mL) and 0.2 μg/mL LPS for 18 h. The mRNA expression of iNOS was detected by real-time RT-PCR. Values are means ± SD (*n* = 3) and significance compared with blank control or LPS treated alone, ^##^ *P* < 0.01 *vs*. blank control group, *P < 0.05, **P < 0.01 *vs*. LPS-treated group. **c** Intracellular NO was detected by confocal laser scanning microscope: (*a*) blank control; (*b*) cells pretreated with LPS; (*c*) cells pretreated with EEDL in the present of LPS; (*d*) cells pretreated with 100 μM L-NAME in the present of LPS. **d** The protein expression of iNOS was measured with Western blot. The optical density was captured by the Image-Pro Plus version 6.0. Values are means ± SD (*n* = 3) and significant difference compared with blank control or LPS treated alone, ^##^ *P* < 0.01 *vs*. blank control group, **P < 0.01 *vs*. LPS-treated group

To estimate how EEDL modulated inflammation responses, we first investigated the effect of EEDL on LPS-induced nitrite accumulation in LPS-induced RAW 264.7 cells. Results in Fig. 4a showed the nitrite concentrated in culture medium was increased 4 times after stimulated by LPS (*P* < 0.01), and a dose-dependent antagonism was observed in EEDL treatment group (*P* < 0.01). Moreover, the effectiveness of the highest concentration was better than aspirin but far less than that of L-NAME. Similarly, confocal scanning micrographs displayed EEDL was a potential anti-inflammation agent by reducing the intracellular NO production stimulated by LPS alone (Fig. 4c).

For the detections of iNOS mRNA and protein expression, EEDL concentration-dependently decreased the abnormal iNOS mRNA (Fig. 4b) and protein (Fig. 4d) (*P <* 0.01 or *P <* 0.05) expression. Especially, administrated with the high dose of EEDL (200 μg/mL) displayed more potently anti-inflammatory effect (inhibitory rate was about 90 %) on reducing the mRNA and protein expressions of iNOS compared with positive control group, pretreated with aspirin.

#### Effect of EEDL on PGE_2_ production, COX-2 mRNA and protein expression

**Fig. 5:**
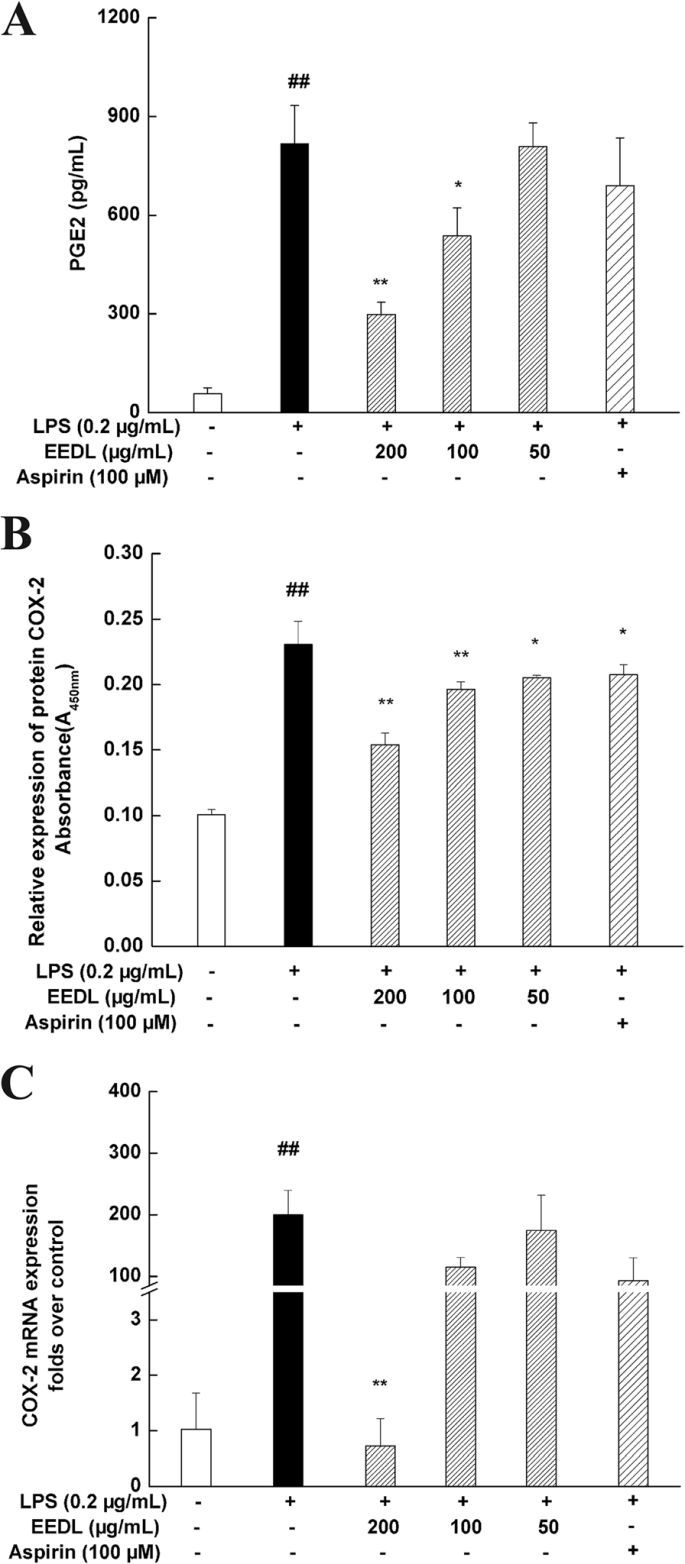
Effects of EEDL on levels of PGE_2_ and COX-2 in LPS-stimulated RAW 264.7 cells. PGE_2_ production (**a**) and COX-2 protein (**b**) and  mRNA expression (**C**) were detected by ELISA and real-time RT-PCR. The protein expression of COX-2 (**b**) was detected by cell-based ELISA. Values are means ± SD (*n* = 6 for ELISA, *n* = 3 for real-time RT-PCR) and significant difference compared with blank control or LPS treated alone, ^##^ *P* < 0.01 *vs*. blank control group, *P < 0.05, **P < 0.01 *vs*. LPS-treated group

PGE_2_ is another important inflammatory mediator. Its production is regulated by COX-2 which catalyzed the metabolism of arachidonic acid. The high concentration of PGE_2_ increases vascular permeability and evokes the inflammatory reaction. As shown in Fig. 5a, EEDL at 200 and 100 μg/mL possessed significant inhibitory activities on LPS-induced PGE_2_ over-production (*P* < 0.05 or *P* < 0.01) and stronger than that of aspirin. For COX-2 mRNA expression (Fig. 5c), a dose-dependent inhibition was observed (200 μg/mL, *P* < 0.01). To evaluate the regulation of EEDL on COX-2 protein expression, cell-based ELISA (Fig. 5b) was performed. Results depicted that EEDL markedly reduced the LPS-induced COX-2 protein over-expression (*P* < 0.05 or *P* < 0.01).

#### Effects of EEDL on IL-6 mRNA expression and protein secretion

**Fig. 6:**
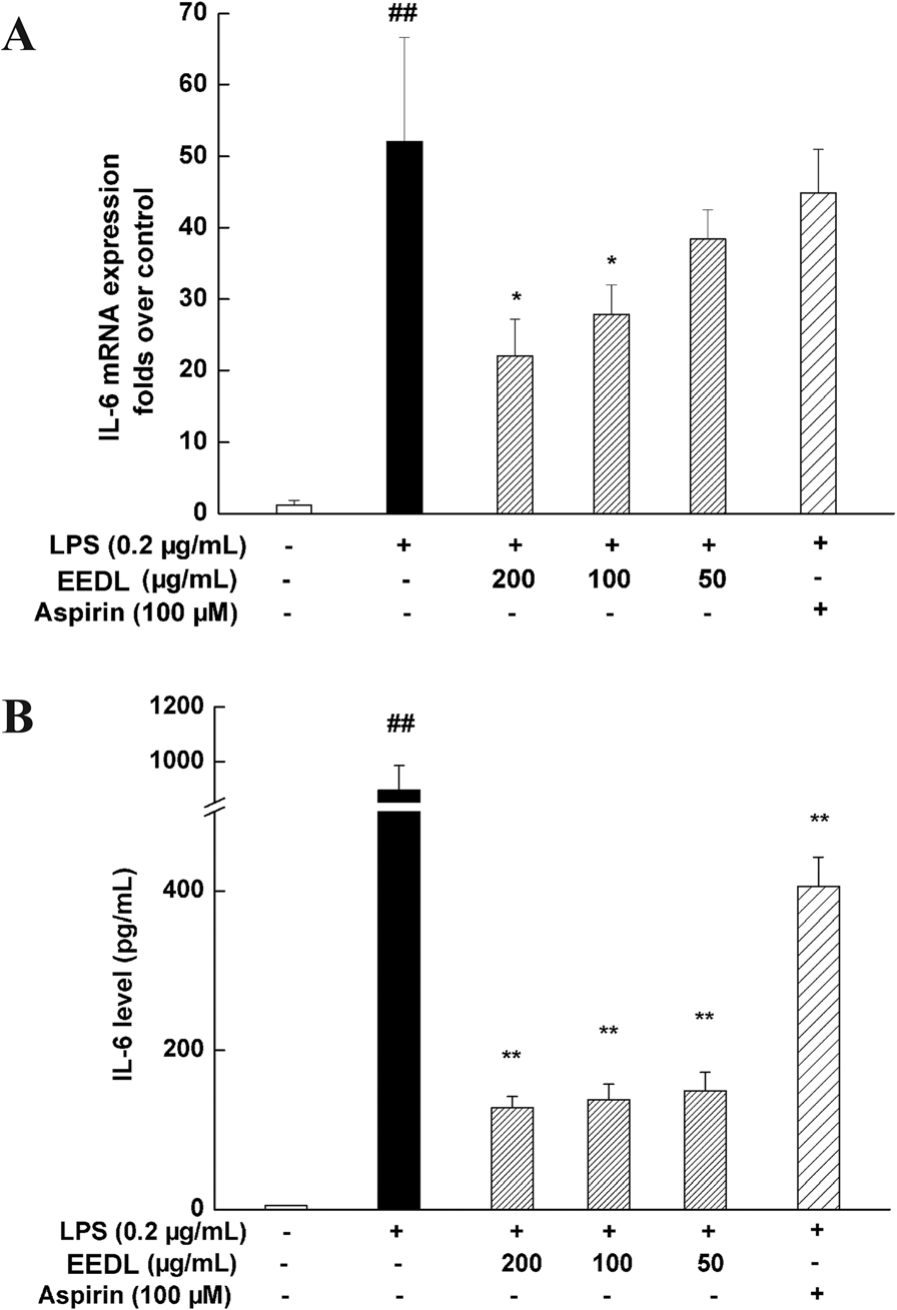
Effects of EEDL on IL-6 mRNA expression and protein secretion in LPS-stimulated RAW 264.7 cells. RAW 264.7 cells were treated with the indicated concentrations of EEDL in the presence of LPS (0.2 μg/mL) for 24 h. The mRNA expression of IL-6 (**a**) was determined by real-time RT-PCR. Values are means ± SD (*n* = 3) and significant difference compared with blank control or LPS treated alone, ^##^ *P* < 0.01 *vs*. blank control group, *P < 0.05 *vs*. LPS-treated group. The protein secretion of IL-6 (**b**) was measured with ELISA. Values are means ± SD (*n* = 6) and significant difference compared with blank control or LPS treated alone, ^##^ *P* < 0.01 *vs*. blank control group, **P < 0.01 *vs*. LPS-treated group

As shown in Fig. 6, the dramatic increase of pro-inflammatory cytokine IL-6 was generally observed following the stimulation with LPS for 24 h. With the intervention of EEDL, however, IL-6 mRNA and protein levels were concentration-dependently decreased (*P* < 0.05 or *P* < 0.01). The inhibitory rate of IL-6 protein secretion was above 50 %.

#### Effects of EEDL on multiple cytokines in LPS-induced RAW 264.7 cells

**Fig. 7:**
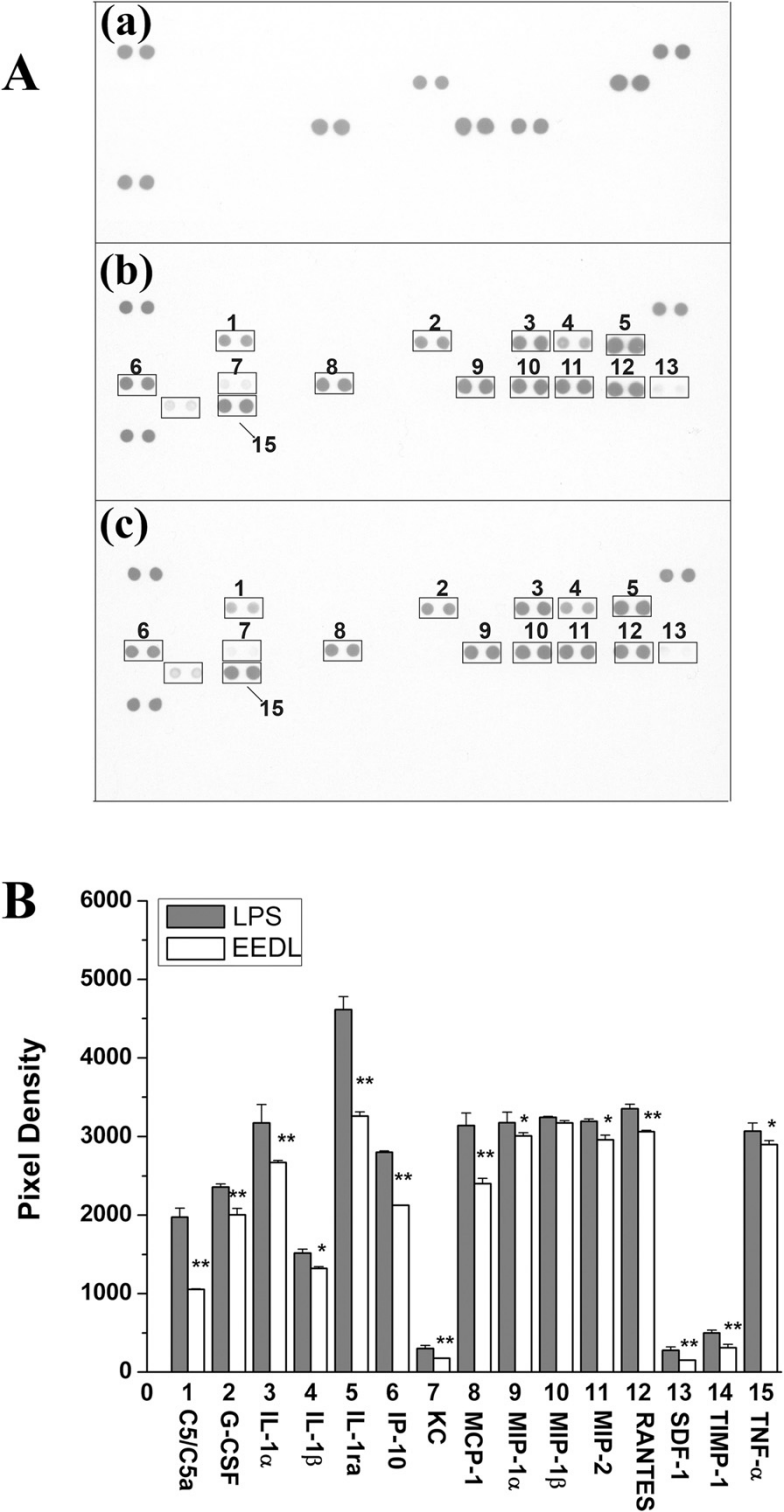
Effects of EEDL on LPS-induced multiple cytokines production in RAW 264.7 cells. **a** Expression of multiple proteins in the acute inflammation was measured by proteome profile array. (*a*) Blank control; (*b*) RAW 264.7 cells were treated with LPS alone; (*c*) RAW 264.7 cells were treated with LPS + EEDL. Numbers marked on the membranes are presented as: “1” C5/C5a; “2” G-CSF; “3” IL-1α; “4” IL-1β; “5” IL-1ra; “6” IP-10; “7” KC; “8” MCP-1; “9” MIP-1α; “10” MIP-1β; “11” MIP-2; “12” RANTES; “13” SDF-1; “14” TIMP-1; “15” TNF-α. **b** Analysis of optical density was captured by the Image-Pro Plus version 6.0. Values are means ± SD and significant difference compared with LPS treated alone, *P < 0.05, **P < 0.01

As shown in Fig. 7, LPS resulted in a burst of 15 inflammation-related proteins in cell lysate, including C5/C5a, G-CSF, IL-1α, IL-1β, IL-1ra, IP-10, KC, MCP-1, MIP-1α, MIP-1β, MIP-2, RANTES, SDF-1, TIMP-1, and TNF-α. However, EEDL treatment (200 μg/mL) notably alleviated the over-expressions of C5/C5a, G-CSF, IL-1α, IL-1β, IL-1ra, IP-10, KC, MCP-1, MIP-1α, MIP-2, RANTES, SDF-1, TIMP-1, and TNF-α (*P <* 0.01 or *P <* 0.05), but with no significant influence on MIP-1β (*P >* 0.05).

#### Effects of EEDL on the DNA-binding activity of NF-κB p65

**Fig. 8:**
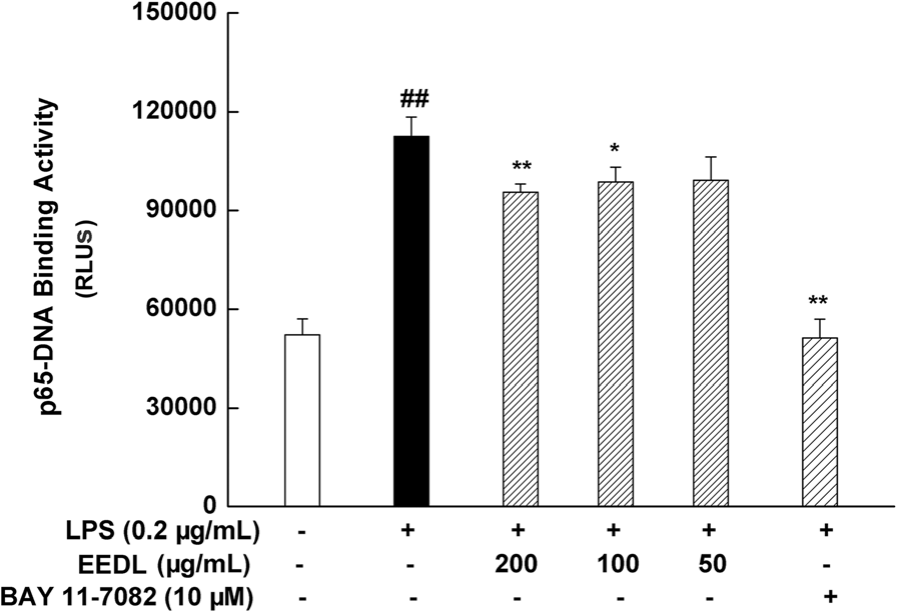
Effects of EEDL on LPS-induced DNA-binding activity of NF-κB p65 in RAW 264.7 cells. RAW 264.7 cells cultured in 6-well plate were pre-treated with EEDL (200, 100 and 50 μg/mL) for 2 h, then the LPS (with a final concentration of 0.2 μg/mL) was added for another 30 min, then the cell lysates were extracted and quantified. The DNA-binding activity of NF-κB p65 was measured by transcription factor assay. Values are means ± SD (*n* = 6) and significant difference compared with blank control or LPS treated alone, ^##^ *P* < 0.01 *vs*. blank control group, *P < 0.05, **P < 0.01 *vs*. LPS-treated group

The activation of NF-κB signaling pathway has been known to play a critical role in the inflammation response. As illustrated in Fig. 8, LPS induced a one-fold increase on the DNA-binding activity of NF-κB p65. After intervened with EEDL for 30 min, however, the over-binding was decreased by EEDL in a dose-dependent manner (*P <* 0.01 or *P <* 0.05).

## Discussion

Mounting evidence highlights that a systemic inflammatory state contributes to the cardiovascular risk [28]. The circulating inflammation mediators take part in the mechanisms of vascular insult and atheromatous changes. As a cardiovascular protective agent, many components of DLP have been proved to have the capacity of anti-inflammation such as gallic acid [29], salvianic acid [30], puerarin [13], daidzin [31], paeoniflorin [32], salvianolic acid B [33], cryptotanshinone [21], and tanshinone IIA [34]. Here, we presented results from the investigation on the efficacy of EEDL on inflammation induced by LPS *in vivo* and *in vitro*.

Studies have shown that LPS injection in mice caused significant inflammatory damage (such as biochemical and histological damage in lung and liver) [23, 35]. In the present study, we used the mice model to investigate anti-inflammation of EEDL. Results showed that EEDL possessed a strong and wide anti-inflammatory effect. In detail, EEDL was effective for antagonizing the over-expression of C5/C5a, G-CSF, I-309, sICAM-1, IL-1ra, IL-6, IP-10, KC, M-CSF, MCP-1, MIP-1α, MIP-1β, MIP-2, RANTES, SDF-1, TIMP-1, and TNF-α (*P <* 0.01), and increasing IL-10 protein level in mice serum (*P <* 0.01). The *in vivo* experiments preliminarily indicated that EEDL might be an effective anti-inflammatory agent.

According to the animal experiments, we tried to further explore the anti-inflammation mechanisms on RAW 264.7 cells, which depicted that treatment of RAW 264.7 cells with EEDL could modulate the iNOS/NO, COX-2/PGE_2_ and cytokine levels by declining the DNA-binding activity of NF-κB p65. iNOS is an important enzyme for mediating inflammatory processes. Generally, NO is at low concentration under physiological conditions which acts as a vasodilator by inhibiting the adhesion of neutrophils to the vascular endothelium [36]. However, large amounts of NO are produced in the inflammatory tissue by the activation of iNOS in both resident tissue cells and infiltrating leucocytes when inflammation was triggered [37]. In this study, we found that the production of NO, intracellular NO, as well as the mRNA and protein expression of iNOS were all up-regulated by LPS, whereas they were attenuated by EEDL treatment. These results indicated that the inhibition of iNOS/NO might be one of the anti-inflammatory targets of EEDL.

In addition to iNOS, COX-2, which is an inducible and isoform enzyme in inflammatory cells [38], is another vital enzyme for mediating inflammatory processes. COX-2 is activated by physiologic stimuli such as inflammation, and is involved in the production of PGs that mediate pain and support the inflammatory process [39]. The PGE_2_, produced by the enzymatic oxidation of arachidonic acid, has been implicated as inflammatory mediators for many years [40]. Additionally, PGs have wide-ranging effects in the body [38]. In this work, we found that EEDL could remarkably decrease the burst production of PGE_2_ based on the detection of EEDL on LPS-induced inflammation.

Moreover, the cell-based ELISA showed that EEDL could dose-dependently antagonize the over-expression of COX-2, which depicted that inhibition of COX-2/PGE_2_ might be another anti-inflammatory target.

Numerous pro-inflammatory cytokines are present in the inflammation process, such as IL-1, TNF, macrophage inflammatory protein (MIP). Firstly, we found that EEDL effectively attenuated the over-expression of IL-6 mRNA and protein induced by LPS in a concentrate-dependent manner. In addition to the ELISA method, protein profile array analysis, a high-throughput way to screen the different acute phase proteins, cytokines, and chemokines in a particular inflammatory process, further indicated that EEDL suppressed LPS-induced inflammation via decreasing multiple pro-inflammatory cytokines such as the common IL-1β, TNF-α, MIP-2.

Studies have demonstrated that NF-κB is involved in the regulation of COX-2 and iNOS expression, namely, blocking the improper NF-κB activation could inhibit the expression of COX-2 and iNOS [41]. Syntheses of cytokines, such as IL-1β, TNF-α, IL-6, are also mediated by NF-κB [42]. Various proteins in NF-κB family play an important role in the defense of the host against certain pathogens [42]. For example, studies have shown that NF-κB p65 plays a role in constitutive IL-6 production in rheumatoid arthritis synovial fibroblasts. In this study, we designed experiment to investigate the DNA-binding activity of NF-κB p65 by transcription factor assay, which showed that the LPS-stimulated NF-κB activation was notably inhibited by EEDL, as indicated by a decreased DNA-binding activity of NF-κB p65.

## Conclusions

We have unraveled a novel pharmacological effect that EEDL effectively suppressed the inflammation induced by LPS in mice. The anti-inflammation of EEDL may be one of the mechanisms of cardiovascular and cerebrovascular disease. Meanwhile, we have preliminarily deciphered the potential molecular mechanisms of EEDL, namely, inhibiting the LPS-induced iNOS/NO, COX-2/PGE_2_ and cytokines levels by decreasing the DNA-binding activity of NF-κB p65. However, we also aware that further researches are needed to obtain more definitive evidence for the deeper exploration.

## Additional file

Additional file 1:
**Cytotoxicity of EEDL on RAW 264.7 cells.** Cells cultured in the 96-well plate for 24 h were incubated with L-NAME (100 μM), aspirin (100 μM) and indicated dilutions of EEDL (200, 100, 50 and 25 μg/mL) in the presence of LPS (0.2 μg/mL) for 20 h. After MTT reagent was added for an additional 2 h, the absorbance was recorded at 570 nm. Values are means ± SD (n=6) from three independent experiments and there is no significant difference compared with LPS treated cells alone. (TIFF 284 kb)
