# Supplementary figures and images for: The anti-inflammatory activities of ethanol extract from Dan-Lou prescription in vivo and in vitro
Source: BMC Complement Altern Med. 2015 Sep 9;15:317. doi: 10.1186/s12906-015-0848-4 (PMC4563854; doi:10.1186/s12906-015-0848-4)

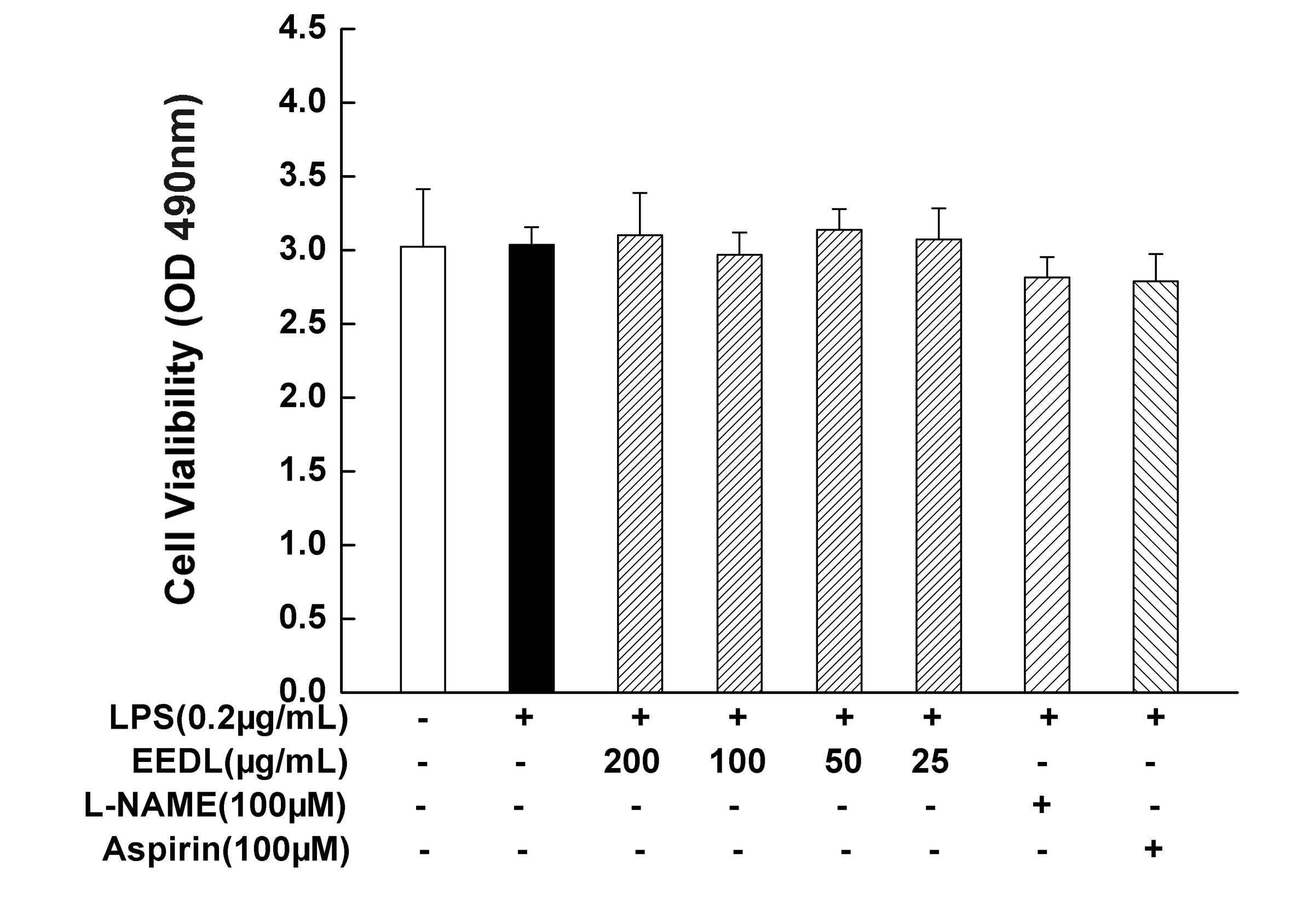

Supplement: Additional file 1: — Cytotoxicity of EEDL on RAW 264.7 cells. Cells cultured in the 96-well plate for 24 h were incubated with L-NAME (100 μM), aspirin (100 μM) and indicated dilutions of EEDL (200, 100, 50 and 25 μg/mL) in the presence of LPS (0.2 μg/mL) for 20 h. After MTT reagent was added for an additional 2 h, the absorbance was recorded at 570 nm. Values are means ± SD (n=6) from three independent experiments and there is no significant difference compared with LPS treated cells alone. (TIFF 284 kb) [file 12906_2015_848_MOESM1_ESM.tiff]
